# Enhanced resistance to *Listeria* infection in mice surviving sepsis: the role of lipid metabolism and myeloid cell reprogramming

**DOI:** 10.3389/fphar.2025.1588987

**Published:** 2025-05-30

**Authors:** Haruki Watanabe, Tengfei Song, Jaewoo Choi, Moses Lee, Kwangmin Choi, Junhwan Kim, Barbara Sherry, Betty Diamond, Yong-Rui Zou, Myoungsun Son

**Affiliations:** ^1^ Institute of Molecular Medicine, Feinstein Institutes for Medical Research, Northwell Health, Manhasset, NY, United States; ^2^ Department of Chemistry, Oregon State University, Corvallis, OR, United States; ^3^ Division of Experimental Hematology and Cancer Biology, Cincinnati Children’s Hospital Medical Center, Cincinnati, OH, United States; ^4^ Institute of Bioelectronic Medicine, Feinstein Institutes for Medical Research, Northwell Health, Manhasset, NY, United States; ^5^ Department of Molecular Medicine, Donald and Barbara Zucker School of Medicine at Hofstra/Northwell, Hempstead, NY, United States

**Keywords:** sepsis, *Listeria* monocytogenes, lipid metabolism, lipid droplets, single-cell RNA sequencing, myeloid cells, lipidomics

## Abstract

**Introduction:**

Immune resilience is the capacity of the immune system to recover from sepsis-induced organ injury and reestablish host defense. While sepsis survivors are often viewed as immunocompromised, recent studies suggest that some may acquire adaptive immune traits that enhance resistance to secondary infections.

**Methods:**

We employed a murine cecal ligation and puncture (CLP) model to study polymicrobial sepsis and subsequent immune responses. Listeria monocytogenes was used as a model intracellular pathogen to assess immune protection. We analyzed myeloid cell phenotypes using single-cell RNA sequencing and evaluated lipid metabolic changes through quantitative RT-PCR, immunohistochemistry, serum cytokine assays, and plasma lipidomics.

**Results:**

Sepsis-surviving mice showed enhanced resistance to Listeria infection. Single-cell RNA sequencing revealed transcriptional reprogramming in splenic CD11b^+^Ly6C^high^ myeloid cells, including downregulation of lipoprotein lipase and lipid efflux genes. CD11b^+^ myeloid cells exhibited increased lipid droplet accumulation, accompanied by elevated serum interferon-gamma (IFN-γ) levels. Plasma lipidomics identified systemic lipid remodeling, with increased phosphatidylserine and decreased phosphatidylinositol and phosphatidylglycerol levels.

**Discussion:**

Our findings suggest that sepsis survival induces lipid metabolic reprogramming in myeloid cells, contributing to enhanced immunity against intracellular pathogens. These insights reveal potential therapeutic targets within lipid metabolic pathways to improve host defense in sepsis survivors.

## Introduction

Sepsis is a complex and life-threatening condition marked by systemic inflammation, tissue damage, and multiple organ failure (Singer et al., 2016; van der Poll et al., 2021). Despite advances in medical care, survivors of sepsis face a significantly elevated risk of recurrent infections, hospital readmission, and long-term mortality (Pool et al., 2018; Cunneen and Cartwright, 2004; Cavaillon et al., 2020; Meyer and Prescott, 2024). While post-sepsis immunosuppression is widely recognized, a subset of survivors demonstrates unexpectedly robust immune control, suggesting the presence of a long-lasting, adaptive immune state known as immune resilience. The acute phase of sepsis involves a hyperinflammatory response followed by a prolonged immunosuppressive state (Ward et al., 2008), which contributes to increased susceptibility to secondary infections. The identification of the factors and pathways that contribute to immune dysfunction in sepsis survivors remains an area of active study. Understanding the cellular and molecular foundations of immune resilience is critical for identifying patients at heightened risk and informing targeted strategies to support durable immune recovery. While it is well-established that sepsis survivors frequently experience persistent immune dysfunction, as well as long-term physical and cognitive impairments that reduce the quality of life for months or even years, some individuals exhibit robust immune resilience (Baudesson de Chanville et al., 2020; Cao et al., 2019; Yanez et al., 2017; Strohl et al., 2024). The concept of immune resilience refers to the ability of the immune system to restore functional equilibrium after severe inflammatory injury (Ahuja et al., 2023; Iskander et al., 2013). Optimal immune resilience is associated with a specific balance between immunocompetence and inflammation, which is linked to favorable immunity-dependent health outcomes (Ahuja et al., 2023). Identifying strategies to reverse sepsis-induced immune suppression and enhance resistance to secondary infections is essential for improving long-term prognosis in sepsis survivors (Monneret et al., 2008; Hotchkiss et al., 2013; Liu et al., 2022; Liu et al., 2019).

A contributing factor in post-sepsis outcomes appears to be the altered function of monocytes and macrophages, particularly in their cytokine responses to pathogens (Delano and Ward, 2016a; Delano and Ward, 2016b). These innate effector cells are central to inflammation and antimicrobial defense and undergo marked changes during and after sepsis (Delano and Ward, 2016a; Delano and Ward, 2016b; Swan et al., 2007; Hortova-Kohoutkova et al., 2020). Baudesson de Chanville and colleagues demonstrated that sepsis induces a two-phase mobilization of monocytes, beginning with an initial phase followed by a delayed and larger-scale mobilization. This second phase is characterized by impaired monocyte function, accumulation in peripheral tissues, and increased susceptibility to infections during recovery (Baudesson de Chanville et al., 2020). A study reanalyzing the dataset of human PBMCs from septic patients revealed a subset of HLA-DR^low^S100A^high^ monocytes with immunosuppressive function, a phenomenon also observed in mouse sepsis (Yao et al., 2023; Reyes et al., 2020).

While most post-sepsis studies focus on responses to extracellular pathogens like *Escherichia coli*, *Klebsiella pneumoniae*, and *Pseudomonas aeruginosa*, the molecular mechanisms contributing to vulnerability to intracellular pathogens remain less well-defined. *Listeria monocytogenes* (Lm), although rare in healthy hosts, can cause severe disease in immunocompromised individuals. Ly6C^high^ monocytes are implicated in Lm dissemination and systemic infection (Jones and D'Orazio, 2017). Once in the bloodstream, Lm rapidly travels to the spleen, where it is internalized by macrophages and dendritic cells (Conlan, 1996). This lifecycle makes Lm highly sensitive to changes in macrophage metabolism, LD accumulation, and antimicrobial programming (Pereira-Dutra et al., 2024). Unlike extracellular bacteria, *Listeria* survival relies on host cell defenses, altering the innate immune response due to prior systemic inflammation.

Lipid metabolism is significantly disrupted during sepsis, leading to altered lipid profiles and metabolic imbalances (O'Neill and Pearce, 2016; Preau et al., 2021; Van Wyngene et al., 2018). Free fatty acids (FFA) in the blood are elevated in sepsis due to the reduced ability of tissues to oxidize them via β-oxidation (Van Wyngene et al., 2018). Sepsis is also associated with intracellular lipid accumulation in the myocardium, resulting from impaired FFA oxidation, which contributes to reduced ATP turnover and myocardial contractile dysfunction (Preau et al., 2021). Dysregulated lipid metabolism can lead to lipotoxicity (Walther and Farese, 2012). One of the critical aspects of lipid metabolism is the formation and accumulation of lipid droplet (LD) within immune cells (Walther and Farese, 2012; Castoldi et al., 2020; van Dierendonck et al., 2022). LDs are intracellular organelles involved in the storage of neutral lipids, such as triacylglycerol (TAG) and cholesterol ester (CE), and their formation is often a cellular response to metabolic stress and inflammation (Walther and Farese, 2012; Zadoorian et al., 2023). LD formation in macrophages is triggered by stimuli such as lipopolysaccharide (LPS) and involves increased uptake of fatty acids and glucose, enhanced TAG synthesis, and inhibition of lipolysis. Importantly, LDs have been shown to participate in immune defense by serving as platforms for antibacterial proteins and modulating cytokine production (Bosch et al., 2020).

Using single-cell RNA sequencing (scRNA-seq) of splenic myeloid cells, we previously found that both C57BL/6J and BALB/c mouse strains, exhibit elevated levels of glycolysis and oxidative phosphorylation in sepsis surviving animals (Watanabe et al., 2024), highlighting the complex metabolic reprogramming associated with sepsis recovery. To elucidate the roles of lipid metabolism in immune resilience and identify dysregulated metabolic pathways, this study further analyzed and focused on LDs in splenic myeloid cells, plasma lipids and secondary Lm infections in sepsis-surviving C57BL/6J mice. We found that the overall monocyte population expanded in the late stages of sepsis, and sepsis-surviving mice exhibited increased resistance to Lm infection. Classical monocytes and a subset of dendritic cell-like monocytes in these sepsis survivors displayed dysregulated lipid metabolism, indicating a significant metabolic shift in the recovery phase.

## Materials and methods

### Mice and sepsis induction

All animal experiments were conducted following institutional guidelines and approved by the Institutional Animal Care and Use Committee (IACUC) of The Feinstein Institutes for Medical Research (Protocol numbers: 2009-048 and 2009-023). Mice were housed in a controlled environment with a 12-h light/dark cycle and provided a rodent diet (Lab Diet, MO, United States) and water.

The CLP model induced sepsis in 8-week-old male C57BL/6J mice (Watanabe et al., 2024; Cuenca et al., 2010; Rana et al., 2018). CLP was performed under isoflurane anesthesia with the local anesthetic bupivacaine 0.25% (one to two mg/kg) administered by subcutaneous injection (s.c.). Mice were given a single dosage of buprenorphine (0.05 mg/kg s. c.) before midline incision. The cecum was isolated and ligated with 4–0 silk sutures below the ileocecal valve and 1 cm from the end of the cecum and then punctured once with a 22-G needle. Following the puncture of the cecum and the extrusion of around 1 mm of feces, the abdominal muscle layer was stitched shut with 6.0 VICRYL^®^ (Ethicon, Raritan, NJ, United States) sutures, and the skin layer was stitched shut using medical-surgical clips. In sham-operated control mice, the cecum was exposed but no ligation nor puncture was performed. Both sham- and CLP-operated mice received one dose of antibiotics (imipenem/cilastatin, 0.5 mg/kg diluted in a 0.9% saline solution) as a part of the resuscitation fluid (total volume of 0.5 mL/mouse, s. c.) and a single dose of sterile saline (0.5 mL/mouse, s. c.). Mice were randomly assigned to CLP or sham treatment. Sample sizes are indicated in figure legends. Animals were checked daily for survival and assessed using the Mouse Grimace Scale twice daily for the first 3 days following surgery, then once daily for up to 7 days. Most experiments produced mortality of 20%–30% within 3–5 days with moderate sepsis severity. Four weeks after surgery, the mice were euthanized.

For the Lm infection study, CLP was performed to induce sepsis in accordance with an established protocol (Song et al., 2023). Twelve weeks post-CLP or sham surgery, mice were infected intravenously with 1 × 10^7^ CFU of Lm. Survival was monitored daily. Approximately 50% of treated mice became moribund under our surgical procedure. For bacterial burden analysis, an independent cohort was infected with 2 × 10^6^ CFU of Lm and euthanized at 72 h post-infection. Livers and spleens were aseptically harvested, weighed, and homogenized in sterile PBS. Serial dilutions of homogenates were plated on Brain Heart Infusion (BHI) agar and incubated overnight at 37°C. Colony-forming units (CFU) were enumerated and normalized per Gram of tissue. CFU data were compared using the Mann–Whitney U test, and results were reported as mean ± SEM.

### Flow cytometry

Cells were resuspended in FACS buffer (PBS supplemented with 2% FBS), stained for different cell surface markers, and then subjected to flow cytometric analysis or cell sorting. Cells (1 × 10^6^ cells per sample) were incubated with Fc block (Rat anti-CD16/CD32, BioLegend) for 15 min at 4°C followed by staining with phycoerythrin (PE)-Cy7-rat anti-CD11b (BD Biosciences, San Jose, CA, United States, 1:200); fluorescein isothiocyanate (FITC)-rat anti-Ly6C (BD Biosciences, 1:100); allophycocyanin (APC)-rat anti- Ly6G (BioLegend; 1:100); anti-mouse CD11 b PE (clone M1/70, eBioscience); Anti-mouse Ly6G APC (clone 1A8, eBioscience); Anti-mouse Ly6C V450 (clone AL-21, BD Biosciences). Following staining the cells were fixed in 1% paraformaldehyde and kept in the dark at 4 °C until analysis. Sample acquisition was performed using LSR Fortessa (BD Biosciences). Data were analyzed with FlowJo software (Tree Star, Inc., Ashland, OR, United States).

### Cell sorting

Splenocytes were isolated from the spleens of a sham- and CLP-operated C57BL/6J mice 4 weeks post-surgery, as previously described (Watanabe et al., 2024). Monocytes were isolated from splenocyte preparations using EasySep™ Mouse Monocyte Isolation Kit (STEMCELL Technologies, Vancouver, Canada). Cells were Fc-blocked with anti-mouse CD16/32 (BioLegend), then stained with PE-Cy7-anti-CD11b, APC-anti-Ly6G and FITC-anti-Ly6C antibodies. CD11b^+^Ly6C^high^Ly6G^−^ populations were sorted using a BD FACSAria II cell sorter (BD Biosciences) under sterile conditions.

### scRNA-seq and analysis

Procedures for isolating splenic monocytes, flow sorting of CD11b^+^Ly6C^high^ cells, scRNA-seq library preparation, sequencing, data processing and cluster prediction have been published (Watanabe et al., 2024). Isolated monocytes were blocked with anti-mouse CD16/32 antibody (BioLegend) for 5 min at 4 °C, then stained with PE-Cy7-anti-CD11b, FITC-anti-Ly6C, and FVD efluor506 alongside various TotalSeq™-B anti-mouse Hashtag antibodies (BioLegend) to distinguish C57BL/6J. After combining cells from two sham-operated or 3 CLP surviving mice, CD11b^+^Ly6C^high^ populations were sorted using the BD FACSAria IIu (BD Biosciences). Cells were combined from two sham-operated and 3 CLP surviving mice and processed on the 10X Chromium Controller (10X Genomics). Gel Bead-in Emulsions were generated with Chromium Next GEM Single Cell 3′Reagent Kits v3.1. RNA and antibody-derived tag libraries were assessed using a High Sensitivity DNA chip (Agilent Technologies). cDNA libraries were sequenced on the Illumina HiSeq to a depth of 26,987 reads per cell for C57BL/6J by Azenta Life Sciences United States).

The raw sequencing data were demultiplexed and aligned with the *Mus musculus* GRCm38 reference genome (Azenta Life Sciences). FASTQ files were further demultiplexed using TotalSeq™ hashtags via the Cell Ranger multi pipeline by BioLegend to clarify mouse-derived data. The scRNA-seq dataset was processed using the Cellenics^®^ community instance (https://scp.biomage.net/) hosted by Biomage (https://biomage.net/). Clusters identified by the Louvain method were visualized at a resolution of 0.8 using UMAP to capture key biological information. Principal components (PCs) were selected based on cumulative variance (80%) Based on 13 clusters, the C57BL/6J strain was further analyzed to compare sham and CLP surviving mice. Statistical tests used in the Seurat were performed with standard statistics tools implemented in R (version 4.23). Differentially expressed genes (DEGs) analyses were performed using Seurat (v.5.0.1). DEGs were filtered by logFC >0.25 and adjusted *P* values of (FDR) < 0.05. Pathway enrichment analysis using Downregulated-DEGs (|log2fold| > 2 and FDR <0.05) in CLP compared to sham (KEGG).

### ELISA assay

To measure interferon-gamma (IFN-γ) levels, mouse serum was collected from sepsis surviving mice 4 weeks post-surgery. Blood was collected via cardiac puncture, and serum was processed by centrifugation for 15 min at 3,000 rpm and 4°C. Serum samples were stored at −80°C until analysis. IFN-γ concentrations in serum were measured using a mouse Proinflammatory 7-plex assay according to the manufacturer’s protocol (Meso Scale Discovery, Rockville, MD, United States). Plates were analyzed on the MS2400 Imager (Meso Scale Discovery). Data were quantified by fitting a standard curve using Meso Scale Discovery Workbench software with default parameters.

### RNA isolation and quantitative RT-PCR

Total RNA was extracted from cells using a Direct-zol RNA Microprep kit (Zymo Research) according to the manufacturer’s instructions. Reverse transcription was performed using the iScript™ cDNA Synthesis Kit (Bio-Rad) with random hexamers. Pre-amplification of low-input cDNA was carried out using TaqMan™ PreAmp Master Mix (Thermo Fisher Scientific) following the manufacturer’s protocol (14 cycles). Quantitative PCR was performed on a LightCycler^®^ 480 II system (Roche) using TaqMan™ Gene Expression Assays (Thermo Fisher Scientific). Quantitative PCR was performed on a LightCycler^®^ 480 II system (Roche) using TaqMan™ Gene Expression Assays (Thermo Fisher Scientific). Target genes included *Lpl* (Mm00434770_m1), *Abca1* (Mm00442646_m1) and, *Abca4* (Mm00492035_m1). Expression values were normalized to Gapdh (Mm99999915_g1). Relative expression was calculated using the 2^−ΔΔCt^ method. All reactions were performed in duplicate or triplicate, and each condition included 5–6 biological replicates (individual mice).

### Immunohistochemistry

One-half of the spleen was embedded in Optimal Cutting Temperature (OCT) compound (Thermo Fisher Scientific), snap-frozen in liquid nitrogen, and stored at −80 °C. Cryosections (10 μm thickness) were obtained using a Leica CM1950 cryostat and collected on Superfrost Plus slides (Fisher Scientific). Sections were fixed in 4% paraformaldehyde for 15 min at room temperature. Sections were then blocked for 1 h in PBS containing 10% normal rat serum (STEMCELL Technologies). Primary staining was performed overnight at 4 °C using Alexa Fluor 488-conjugated anti-mouse CD11b antibody (BioLegend, clone M1/70; 1:200 dilution in 2% normal rat serum). Slides were then washed and stained with LipidTOX™ Deep Red or LipidTOX™ Green neutral lipid stains (Thermo Fisher Scientific, 1:500 dilution in PBS) for 30 min at room temperature in the dark. All slides were mounted with Dako Fluorescence Mounting Medium (Dako) and sealed with coverslips.

Confocal images were acquired with a Zeiss LSM900 microscope using Airyscan 2 technology. Z-stacks were collected with ×20 and ×60 oil immersion objectives at Nyquist rates. Imaging parameters (laser intensity, gain, pinhole size) were consistent across experimental groups. Images were captured with Zen Blue software and exported as maximum intensity projections. Quantitative analysis was performed using Imaris software (Bitplane, v9.8), rendering CD11b^+^ cell volumes in 3D, and colocalization with LipidTOX was analyzed using the “Surface-Surface Colocalization” module. Total colocalized voxel volume per cell was calculated and averaged across fields. A blinded investigator analyzed at least three spleen sections per mouse and three regions per section.

### Untargeted plasma lipidomics analysis

Blood was collected via cardiac puncture after 2 h of fasting from CLP and sham-operated mice at 4 weeks post-surgery. Plasma samples from heparin-treated blood were centrifuged at 1,500 x g for 10 min at 4°C. The supernatant was collected and stored at −80°C. Frozen plasma samples (20 µL per mouse) were thawed on ice and extracted using 480 µL of lipid extraction solvent consisting of methylene chloride:methanol:isopropanol (25:10:65, v/v/v) supplemented with 0.1% butylated hydroxytoluene (BHT) as an antioxidant. Samples were vortexed for 30 s, followed by centrifugation at 13,000 rpm for 10 min at 4 °C. A 98 µL aliquot of the organic phase was transferred into LC-MS/MS vials and spiked with 2 µL of SPLASH^®^ Lipidomix^®^ internal standard mixture (Avanti Polar Lipids). Lipidomic profiling was performed using ultra-high-performance liquid chromatography (UHPLC) on a Waters Acquity UPLC CSH C18 column (2.1 mm × 100 mm, 1.7 µm particle size) coupled to a TripleTOF 5,600 mass spectrometer (AB Sciex) operated in information-dependent acquisition (IDA) MS/MS mode. LC-MS conditions were adapted from Choi et al. (Choi et al., 2015). In positive ion mode, mobile phase A consisted of 60:40 acetonitrile:water with 10 mM ammonium formate and 0.1% formic acid; mobile phase B consisted of 90:10 isopropanol:acetonitrile with the same additives. In negative ion mode, 10 mM ammonium acetate was substituted as the buffer modifier. To control inter-batch variability and assess instrument stability, we included the NIST Standard in each analytical run alongside mouse plasma, using identical extraction, handling, and MS acquisition protocols. Peak areas were normalized to the appropriate internal standard for each lipid subclass. Absolute concentrations were estimated using molar response factors derived from the NISTIR 8185 and LIPID MAPS databases. Lipid nomenclature was standardized according to the LIPID MAPS classification (Bowden et al., 2017) (Supplementary Table S1; Supplementary Figure S1). After normalization, data were log-transformed and Pareto scaled for statistical analysis using MetaboAnalyst (v6.0). Partial Least Squares Discriminant Analysis (PLS-DA) was performed to visualize global metabolic differences between groups.

### Statistical analysis

All statistical analyses were performed using GraphPad Prism version 9.5.1 (GraphPad Software, La Jolla, CA, United States). Data are presented as mean ± standard error of the mean (SEM). Statistical significance was determined using a threshold of p < 0.05. For comparisons between two groups, unpaired two-tailed Student’s t-tests were applied when data were normally distributed. For non-normally distributed data, the Mann–Whitney U test was used. For multiple group comparisons, one-way ANOVA followed by Tukey’s *post hoc* test was employed. Survival was evaluated using the log-rank test. In all cases, sample sizes and exact p-values are reported in the figure legends.

## Results

### Resistance to secondary infections with *Listeria monocytogenes* in mice surviving sepsis

**FIGURE 1:**
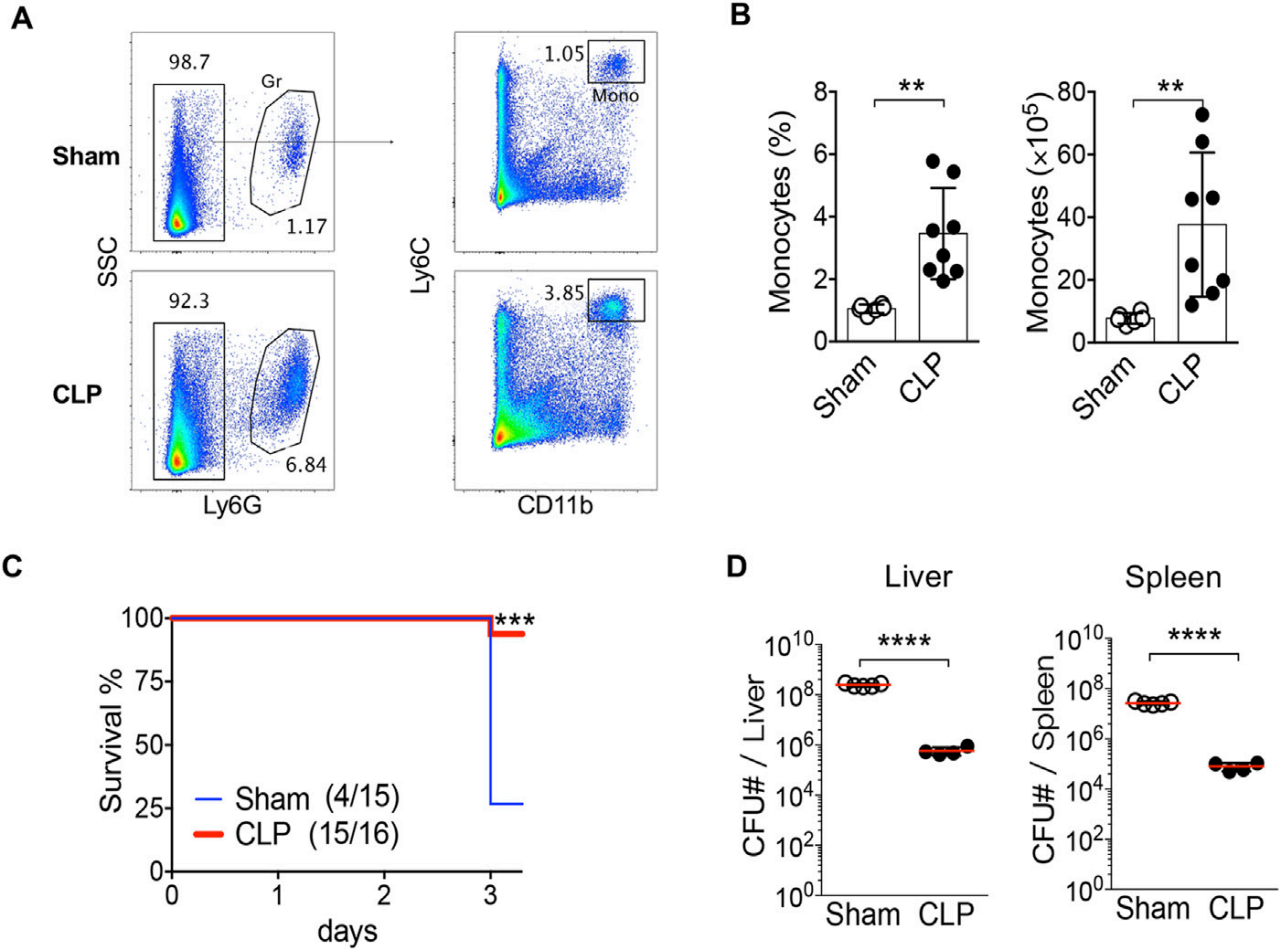
Resistance to Secondary Infection and Inflammatory Response. **(A)** Flow cytometry analysis of splenic granulocytes (Gr) and monocytes (Mono) revealed significant differences between sham and CLP mice. **(B)** Statistical analysis showed increased percentages and total numbers of splenic monocytes in CLP mice compared to sham controls, indicating an altered immune cell population post-sepsis. Data represent mean ± SEM. Group comparisons were analyzed using unpaired two-tailed t-tests. **(C)** Kaplan-Meier survival curves illustrated that CLP mice had a significantly higher survival rate following Lm infection than sham controls, with a survival percentage of 94% in CLP versus 27% in sham (p < 0.001, log-rank test). **(D)** Bacterial load assessments in the liver and spleen 3 days post-infection demonstrated significantly lower colony-forming units (CFU) in CLP mice, indicating better bacterial clearance. **Each dot represents an individual mouse; bars indicate mean ± SEM. Statistical comparisons were performed using Mann–Whitney U test (**p < 0.0001). All experiments were repeated independently at least twice with consistent results.

Previous research indicates that the population of splenic monocytes becomes both heterogeneous and significantly expands after sepsis, showcasing increased phagocytic activity (Delano and Ward, 2016a; Delano and Ward, 2016b). In particular, CD11b^+^Ly6C^high^Ly6G^−^ monocyte-derived myeloid-derived suppressor cells (MDSC) have been shown to proliferate in murine sepsis models. These cells experience long-term metabolic reprogramming, which includes impaired mitochondrial respiration, enhanced aerobic glycolysis, and upregulation of granule protease gene expression (Watanabe et al., 2024). Sustained myelopoiesis represents another characteristic of hematologic remodeling post-sepsis, as there are increased numbers of circulating monocytes and granulocytes noted for extended durations following the resolution of infection (Song et al., 2023). Consistent with previous findings, flow cytometry analysis revealed substantial shifts in splenic myeloid cell populations, including an increase in CD11b^+^Ly6G^+^ granulocytes and CD11b^+^Ly6C^high^ monocytes in CLP mice (Figures 1A,B). Our group previously showed sustained elevation of peripheral monocytes and granulocytes at 2-, 4-, 10-, and 12-week post-CLP, with some animals maintaining higher levels of circulating myeloid cells up to 7 months following the initial insult (Song et al., 2023). Additionally, the total number of monocytes was significantly higher in CLP mice (Figure 1B). Given that Ly6C^high^ monocytes, rather than granulocytes, are linked to the dissemination and systemic spread of Lm, we focused our mechanistic analysis on this subset.

To assess long-term immune responses, a separate cohort of mice was challenged with Lm at 12 weeks post-CLP or sham surgery. We challenged CLP-survivor mice with Lm, a model intracellular pathogen primarily cleared by myeloid cells such as macrophages. Lm was selected because of its well-characterized reliance on innate cell-intrinsic defense mechanisms and monocyte/macrophage-mediated clearance, making it an ideal model to assess immune resilience. Mice were infected intravenously via the tail vein and monitored daily for survival. CLP mice displayed improved resistance to secondary Lm infection, exhibiting a significantly higher survival rate compared to sham controls 3 days post-infection (Figure 1C). Furthermore, the bacterial load in the liver and spleen of the surviving CLP mice was significantly lower than that observed in the sham controls on day 3 (Figure 1D), suggesting improved bacterial clearance.

### Accumulation of LD in the spleens and elevated serum levels of IFN-γ in sepsis-surviving mice

**FIGURE 2:**
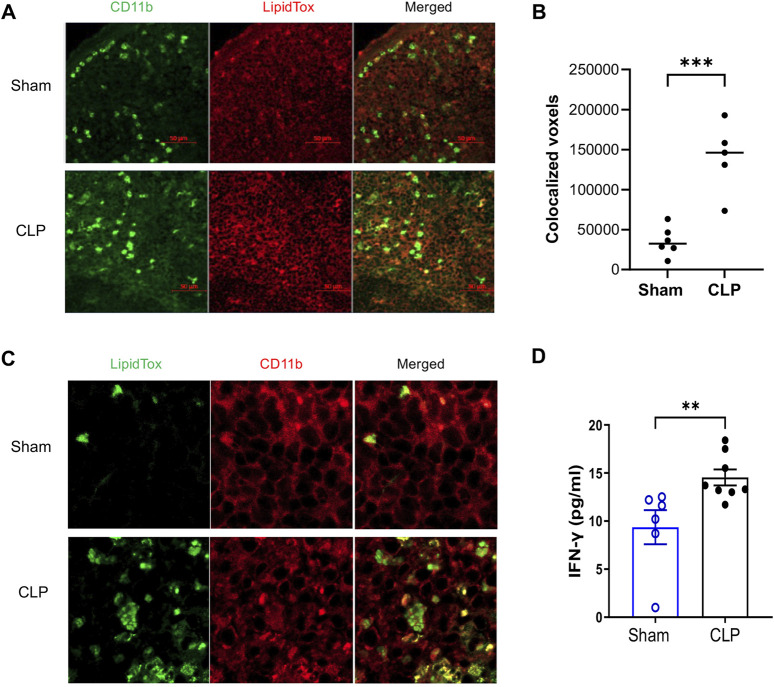
Accumulation of Lipid Droplet (LD) in CD11b^+^ Myeloid Cells from Sepsis-Surviving Mice Compared to Sham Controls. Confocal microscopy images show LD accumulation in CD11b+ myeloid cells from CLP (n = 5) and sham controls (n = 6). **(A)** Representative images display CD11b staining (green), LD (LipidTox Deep Red), and merged channels at ×20 magnification. Scale bar = 50 µm. **(B)** Quantitative analysis of LD accumulation was performed by calculating the colocalized voxel volume of CD11b and LipidTox signals across at least three fields per spleen, averaged per mouse. CLP mice showed significantly increased LD accumulation compared to sham controls (p < 0.001, unpaired two-tailed t-test). Bars represent mean ± SEM. **(C)** High-resolution images (60× with 2× zoom) illustrate extensive LD accumulation in CD11b^+^ cells from CLP mice, confirming qualitative differences in LD abundance. Representative images are consistent across multiple fields and independent replicates. All staining and imaging were performed under identical acquisition settings and analyzed in a blinded manner. **(D)** Higher IFN-γ levels in the serum of sepsis survivors 4 weeks post-CLP surgery were observed compared to sham control mice (n = 6-8 mice per group, Mann–Whitney test). Results are presented as the mean ± SEM.

Given the established role of LD in modulating macrophage inflammatory and antimicrobial function (Bosch et al., 2020; Bosch et al., 2021), we hypothesized that increased LD accumulation in myeloid cells might contribute to the immune resilience observed in sepsis survivors. To test this, we examined LD in splenic myeloid cells using confocal microscopy. LipidTOX staining revealed marked increases in LD content within CD11b^+^ myeloid cells from CLP mice compared to sham controls (Figure 2A). Quantitative analysis confirmed both the number and total volume of LD colocalized with CD11b signal were significantly elevated in CLP mice (Figure 2B). Notably, high-magnification imaging showed that individual CD11b^+^ cells from CLP mice exhibited substantial LD accumulation per cell (Figure 2C). To observe cytokine levels indicating an immune resilience mechanism, we measured IFN-γ serum levels 4 weeks post-surgery (Figure 2D). CLP survivors exhibited higher IFN-γ levels compared to control mice, suggesting an enhanced response to pathogens (Sica and Mantovani, 2012).

### Changes in gene expression in CD11b^+^Ly6C^high^ myeloid cells

**FIGURE 3:**
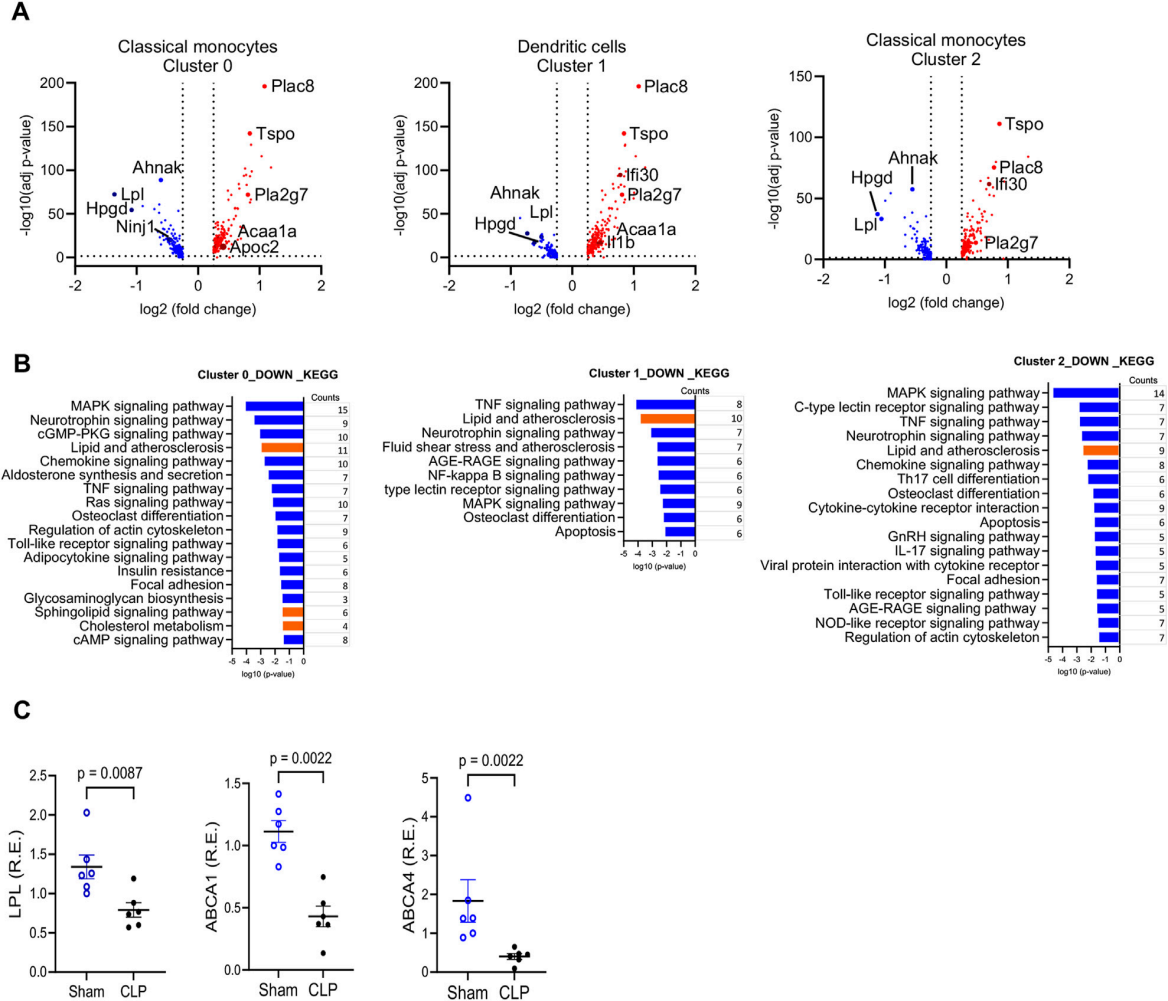
Gene Expression Changes in CD11b^+^Ly6C^high^ Myeloid Cells from Sepsis Survivors Compared to Sham Controls. **(A)** Volcano plot showing differentially expressed genes (DEGs) in CD11b^+^Ly6C^high^ myeloid cells from CLP versus sham mice. DEGs were defined as |log_2_ fold change| > 2 with FDR <0.05. The y-axis shows the–log_10_-adjusted p-values; upregulated genes in CLP are shown in red, downregulated in blue, and selected genes of interest are annotated. **(B)** KEGG pathway analysis of the top downregulated biological processes in clusters 0, 1, and 2 derived from scRNA-seq of splenic myeloid cells. Pathway enrichment was based on DEGs with adjusted p-value <0.05 and visualized using log-transformed p-values. Lipid-related pathways are highlighted in red. **(C)** Quantitative real-time PCR validation of selected DEGs in flow-sorted CD11b^+^Ly6C^high^ myeloid cells from sham and CLP mice at 4 weeks post-surgery. Genes tested included *Lpl*, *Abca1*, and *Abca4*. **Data are presented as mean ± SEM; n = 6 (sham), n = 5–6 (CLP). Group comparisons were performed using unpaired two-tailed t-tests (*p < 0.05, p < 0.01). Each qRT-PCR was run in technical duplicate and repeated in at least two independent experiments.

To identify the biological pathways associated with enhanced bactericidal activity of myeloid cells post sepsis, we analyzed the published scRNA-seq on splenic CD11b^+^Ly6C^high^ myeloid cells from sham-operated and CLP mice (Watanabe et al., 2024). As we previously reported, among 13 clusters, classical monocytes were identified in clusters 0, 2, 3, and 6, and dendritic cells were in clusters 1 and 9 (Supplementary Figure S2A). The frequency maps showed no significant difference in these clusters in C57BL/6J. Cluster 0 shows 15% compared to 14%, cluster 2 shows 13% versus 13%, cluster 3 shows 13% compared to 14%, cluster 6 shows 5.5% versus 4.4%, cluster 1 shows 12% versus 13% and cluster 9 shows 5% compared to 4.3%. Thus, approximately 60% of CD11b^+^Ly6C^high^ classical monocytes and dendritic cells exist in sham controls and CLP mice (Supplementary Figure S2B) (Watanabe et al., 2024). Within these classical monocytes and dendritic cell subpopulations (clusters 0, 1 and 2), the differential expression of genes in the CLP group showed altered lipid metabolism-related genes (Figure 3A; Supplementary Figure S3). Genes such as Lipoprotein Lipase (*Lpl*), AHNAK Nucleoprotein (*Anhak*) and 15-Hydroxyprostaglandin Dehydrogenase (*Hpgd*)), which are involved in lipid metabolism, were significantly downregulated in the CLP group. LPL hydrolyzes TAG in lipoproteins into free FAs, which cells can take up for energy production or storage (Eckel, 1989; Goldberg, 1996). The reduced expression of LPL in CLP mice suggests a compromised ability to process and utilize FFA, contributing to lipid accumulation within the cells (Loving et al., 2021). AHNAK regulates cytoskeletal actin filament organization and calcium signaling (Hohaus et al., 2002), and AHNAK-deficient mice exhibited reduced fat accumulation in the liver and decreased serum TAG levels when provided with a normal chow or high-fat diet (Kim et al., 2021). HPGD is responsible for the metabolism of prostaglandins (Hughes et al., 2008). Figure 3A also showed other metabolism-related genes, such as Placenta Specific 8 (PLAC8), Translocator Protein (TSPO), and Phospholipase A2 Group VII (PLA2G7), were upregulated in the CLP model. Moreover, KEGG pathway analysis highlighted downregulated pathways involved in lipid metabolism in several clusters of myeloid cells from sepsis survivors (Figure 3B). We found significant downregulation of lipid efflux genes (*Abca1*, *Abca4*, and *Abcg1*) in most clusters of sepsis survivors. Quantitative RT-PCR confirmed some of the transcripts (*Lpl, Abca1and Abca4)* from isolated CD11b^+^Ly6C^high^ myeloid cells (Figure 3C). The downregulation of lipid efflux genes suggests an impaired ability of these cells to export excess lipids, potentially leading to lipid accumulation.

### Analysis of plasma lipidomics

**FIGURE 4:**
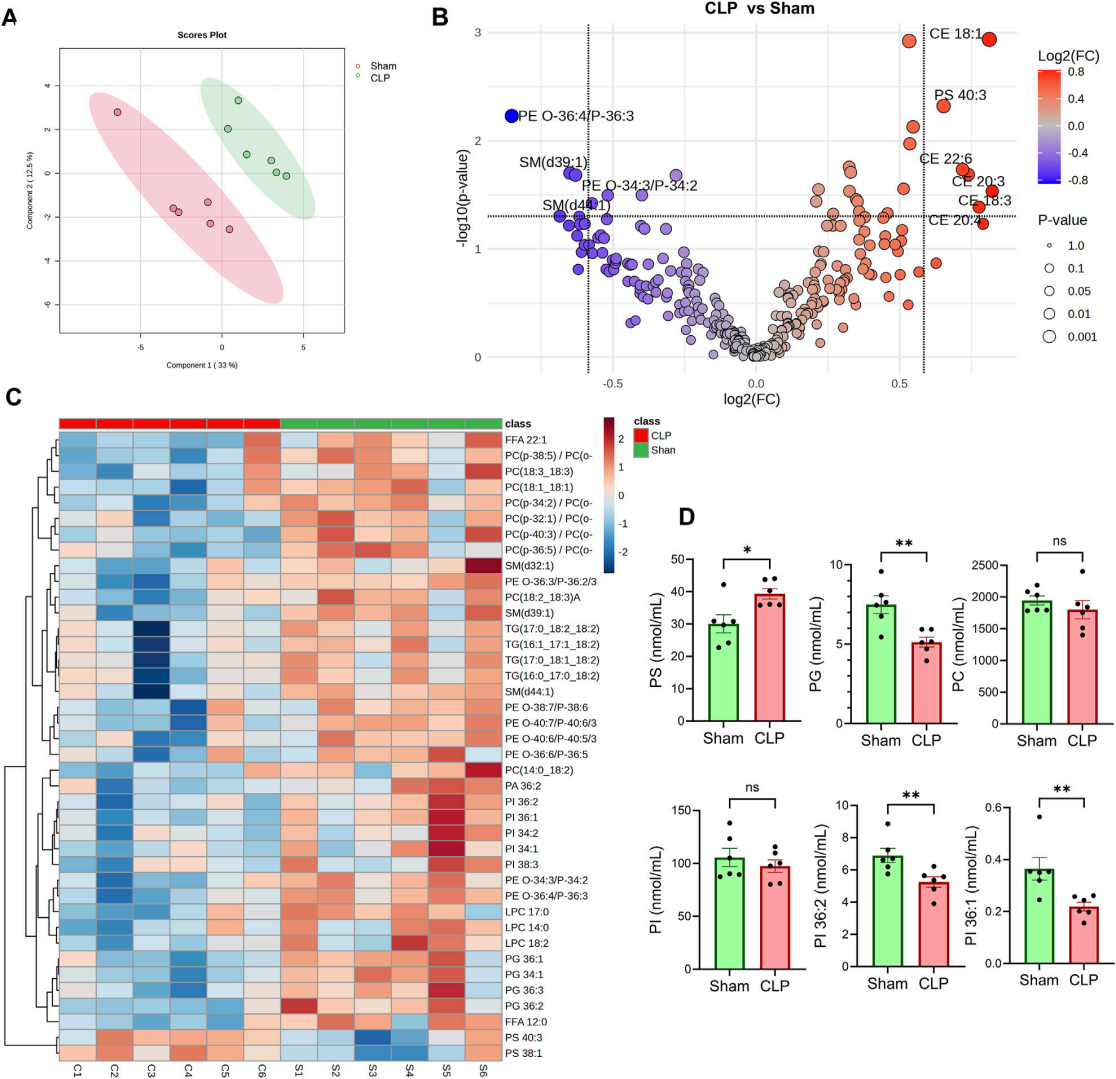
Lipidomic Profiling in CLP and Sham Groups. **(A)** Partial least squares discriminant analysis (PLS-DA) of plasma lipidomic profiles from CLP and sham mice (n = 6 per group). **(B)** Volcano plot displays lipid species that are differentially abundant between CLP and sham groups. **(C)** Heatmap visualization of the relative abundance of annotated lipid species across individual samples. TG, triacylglycerols, DG, diacylglycerols, and MAG, monoacylglycerols. PC, phosphatidylcholine; LPC, lyso-PC; PI, phosphatidylinositol; PS, phosphatidylserine; LP, PE, phosphatidylethanolamine; SM, sphingomyelin; FFA, free fatty acid. **(D)** Quantitative comparison of specific lipid classes, including total phosphatidylserine (PS), phosphatidylcholine (PC), c, and individual PI species (PI 36:1 and PI 36:2). **Data represent mean ± SEM. Statistical comparisons were performed using unpaired two-tailed t-tests (*p < 0.05, p < 0.01; ns = not significant).

To evaluate systemic lipid alterations associated with sepsis recovery, we performed untargeted plasma lipidomics on CLP-surviving and sham-operated mice. Data were normalized to internal class-specific lipid standards and total protein content prior to analysis (Supplementary Table S2). Partial least squares discriminant analysis (PLS-DA) revealed a clear separation between the CLP and sham groups (Figure 4A). Several phospholipid species, including phosphatidylglycerol (PG 36:2, 34:1), phosphatidylinositol (PI 36:1), phosphatidylcholine (PC 36:5, 36:6), sphingomyelin (SM 39:1, 44:1), and phosphatidylethanolamine (PE 36:4, 36:3), were significantly decreased in the CLP group (Figure 4B). In contrast, there were significant increases in cholesterol ester (CE) species, including CE 18:1, CE 22:6, CE 20:3, CE 20:4, and CE 18:3 (Figure 4B). These lipid species are known to localize within lipid droplets. Phosphatidylserine (PS) species such as PS 40:3 and PS 38:1 were markedly elevated in sepsis survivors (Figures 4B–D). PG was significantly reduced, consistent with mitochondrial membrane remodeling (Figure 4D). While total PI remained unchanged, species-specific analysis revealed a significant decrease in PI 36:2 and PI 36:1, which are lipids critical for plasma membrane dynamics and phagocytic signaling. These shifts did not extend to PC, which remained statistically unchanged (Figure 4D).

## Discussion

We present evidence indicating that lipid profiles in plasma and heterogeneity of CD11b^+^Ly6C^high^ myeloid cells are associated with differential expression of genes involved in lipid metabolism in mice surviving sepsis. We also found that mice post-sepsis exhibited enhanced resistance to Lm infection and improved tissue bacterial clearance. The potent mechanism may stem from a lack of lipid efflux, leading to LD accumulation in myeloid cells in sepsis survivors. To our knowledge, this is the first study to demonstrate that CD11b^+^ myeloid cells from sepsis survivors undergo durable lipidomic and transcriptional reprogramming that correlates with enhanced bactericidal activity.

ScRNA-seq identified specific subpopulations of myeloid cells with reduced lipid efflux and glycolipid metabolism gene expression (Figure 3). Notably, there were no significant changes in genes involved in lipid storage or LD biosynthesis, suggesting that LD accumulation might be related to lack of lipid efflux. We also have observed reduced LPL expression in the CLP mice. LPL is reported to be associated with LD accumulation (Loving et al., 2021). In addition, the decreased expression of lipid efflux genes likely impairs lipid transfer from monocytes to plasma, contributing to the observed reductions in plasma CE levels. LD is commonly associated with proinflammatory macrophage phenotypes, which may enhance resistance to secondary infection. In line with previous studies linking dysregulated lipid metabolism to lipotoxicity and organ dysfunction in sepsis, our findings show altered lipid profiles and disrupted metabolic pathways in sepsis survivors, with downregulation of lipid efflux genes appearing to be a central mechanism of LD accumulation. Furthermore, the downregulation of HPGD may result in prolonged inflammation due to the decreased inactivation of pro-inflammatory prostaglandins, exacerbating the systemic inflammation characteristic of sepsis (Parent et al., 2006; Murata et al., 1997). Conversely, upregulated genes in the CLP model such as PLAC8, TSPO, and PLA2G7 also can modulate metabolism and monocyte functions. PLAC8 enhances the activation of the ERK pathway, promotes monocyte proliferation, and activates macrophages (Mao et al., 2021; Kaistha et al., 2016; Sun et al., 2023; Zhang et al., 2024). TSPO is involved in mitochondrial function and steroid synthesis (Batarseh and Papadopoulos, 2010; Rupprecht et al., 2010). PLA2G7 enhances eicosanoid production (Lv et al., 2021; Candels et al., 2022). These differential gene expressions observed in sepsis survivors may strengthen the capacity of myeloid cells to respond to infections, especially for Lm (Michel and Bakovic, 2007; Shin et al., 2008). We previously reported that CLP enhances the phagocytosis of bacterial particles by CD11b^+^Ly6C^high^ myeloid cells in C57BL/6J mice compared to sham controls (Watanabe et al., 2024). This increase in phagocytic capacity was thought to be mediated by Fcγ receptors (Watanabe et al., 2024). Enhanced phagocytosis in CLP mice may improve survival against secondary Lm infections.

Lm was selected for this study due to its well-established utility in investigating macrophage-mediated innate immunity and intracellular bacterial clearance. Because *Listeria* relies heavily on cell-intrinsic host defense mechanisms, it provides a stringent model for evaluating immune competence following sepsis-induced injury. It is important to note that our experiments assessed resistance to *Listeria* specifically in CLP survivors. Our findings regarding lipid profiles in myeloid cells may contribute to strategies for preventing secondary infections, particularly from Lm, which preferentially replicates in splenic macrophages by utilizing host metabolites. Once internalized, *Listeria* replication may be restricted by LD, which is thought to support antimicrobial activity through the recruitment of immune proteins and the facilitation of both local and systemic metabolic responses to infection. While our study is focused on Lm, the lipid metabolic rewiring and enhanced immune function observed here may extend to other intracellular pathogens that engage similar host pathways.

**FIGURE 5:**
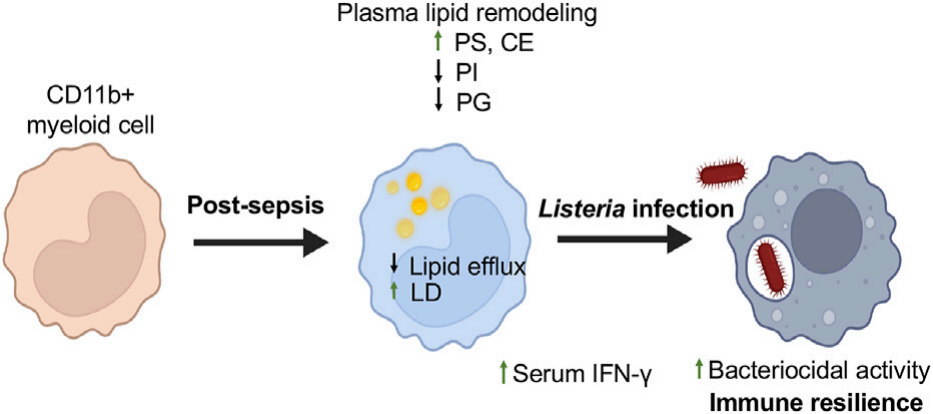
Myeloid Cell Reprogramming Enhances Resistance to Secondary *Listeria* Infection in Sepsis Survivors. This schematic illustrates the immunometabolic adaptations in myeloid cells of mice surviving sepsis that contribute to enhanced resistance against secondary *Listeria monocytogenes* infection. Following CLP-induced sepsis, myeloid cells undergo transcriptional and lipid metabolic reprogramming. These changes include downregulation of lipid efflux genes, increased lipid droplet (LD) accumulation, and shifts in systemic lipid profiles, including elevated phosphatidylserine (PS) and decreased phosphatidylinositol (PI) and phosphatidylglycerol (PG) species. Upon re-challenge with *Listeria*, these trained myeloid cells exhibit enhanced bacterial clearance, reduced organ bacterial burden, and improved host survival, reflecting a state of immune resilience. This model illustrates how targeted lipid remodeling in innate immune cells may influence host defense long after sepsis recovery. Created using Biorender.com.

Lipidomic analysis revealed a distinct plasma lipid signature in sepsis survivors, characterized by elevated PS and reduced levels of PI and PG. PS is associated with efferocytosis and anti-inflammatory signaling, and its increase suggests that macrophages in sepsis survivors retain a capacity for anti-inflammatory and phagocytic function (Martin et al., 2014). PI is a precursor for intracellular signaling lipids like PIP2 and PIP3, which are essential for phagosome maturation (Desale and Chinnathambi, 2021). A reduction in PI species may alter these intracellular signaling pathways, potentially enhancing macrophage sensitivity to pathogens and contributing to improved *Listeria* clearance observed in our CLP model, highlighting lipid-mediated immune resilience. PG depletion may affect membrane structure and mitochondrial dynamics, influencing its metabolism, which is a characteristic of trained immunity. CEs are known to localize within LD. We observed increased LDs in splenic CD11b^+^ myeloid cells of sepsis survivors, along with elevated plasma levels of CE, which suggests a shift in lipid storage and usage that may support immune training. These findings collectively suggest that lipidomic reprogramming contributes to both improved pathogen control and inflammation resolution in the post-sepsis state. Upon exposure to Lm, these reprogrammed myeloid cells display improved bacterial clearance, reduced organ bacterial burden, and increased host survival, indicating a state of immune resilience (Figure 5). Mechanisms may involve insufficient lipid efflux, resulting in LD buildup in myeloid cells and elevated serum levels of IFN-γ in mice that have survived sepsis. The accumulation of LD may amplify early innate immune responses, including IFN-γ modulation and viral clearance mechanisms, as previously demonstrated (Monson et al., 2021).

This study has several limitations. First, our findings are based on a single murine model and focused on *Lm*, an intracellular pathogen, which may limit the generalizability of the results to other types of infections or clinical sepsis contexts. While *Listeria* provides a robust model for studying macrophage-intrinsic immunity responses, future studies should incorporate extracellular pathogens (e.g., *E. coli*, *Klebsiella*) to evaluate whether the observed immune resilience extends to broader infectious challenges. Second, although we identified significant transcriptional and lipidomic changes in myeloid cells, direct mechanistic evidence linking lipid droplet accumulation to enhanced bacterial clearance remains incomplete. Functional validation such as LD^high^ vs LD^low^ macrophage killing assays and pharmacological modulation of lipid metabolism will be critical in follow-up studies. It is essential to define the temporal and cell-sepcific dynamics of LD formation during sepsis recovery. It is also possible that epigenetic reprogramming of myeloid cells, metabolic shifts beyond lipid storage (e.g., increased glycolysis or mitochondrial respiration), or persistent alterations in cytokine receptor signaling contribute to the heightened immune response observed in sepsis survivors. Finally, while plasma lipidomic shifts in PS, CE were significantly high in sepsis surviving mice, we did not assess their tissue-specific effects or determine their source. Future studies are needed to determine the functional impact of these lipid changes on macrophage bactericidal capacity, particularly in relation to LD content and the manipulation of specific lipid metabolic pathways. Ultimately, validating these lipidomic and transcriptomic findings in additional models and testing their causality through targeted functional approaches will be essential to advancing translational insights into post-sepsis immune resilience.

In conclusion, our findings indicate that sepsis survivors exhibit LD accumulation and altered lipid metabolism in myeloid cells, which may contribute to enhanced immune resilience against Lm secondary infections. Elevated lipid storage capacities and altered lipid profiles contribute to the improved immune response against Lm observed in sepsis survivors. This metabolic shift may be influenced by the vagus nerve, which modulates inflammation in sepsis through the cholinergic anti-inflammatory pathway (Rana et al., 2018; Kurata-Sato et al., 2024; Tracey, 2007). Investigating the relationship between neural pathways and LD accumulation in the context of sepsis could prove beneficial.

## Data Availability

The scRNA-seq data are available in the GEO database at https://www.ncbi.nlm.nih.gov/geo/query/acc.cgi?acc=GSE249839, reference number GSE249839. The raw lipidomics data generated and analyzed during this study are in the Supplementary Tables. The data supporting the finding of this study have been deposited in the Dryad Digital Repository: 10.5061/dryad.0p2ngf2cj. Further inquiries can be directed to the corresponding authors.

## Abbreviations

CLP, Cecal Ligation and Puncture; PBMC, peripheral blood mononuclear cell; FA, free fatty acid; LD, Lipid Droplet; TAG, Triacylglycerols; CE, cholesterol ester; DAG, Diacylglycerols; Lm, *Listeria* monocytogenes; scRNA-seq, Single-cell RNA sequencing; LPL, lipoprotein lipase; ABCA1, ATP-binding cassette transporter A1; ABCG, ATP-binding cassette transporter G1; HPGD, 15-Hydroxyprostaglandin Dehydrogenase; PLAC8, Placenta Specific 8; TSPO, Translocator Protein; PLA2G7, Phospholipase A2 Group VII; PS, phosphatidylserine; PI, phosphatidylinositol; PG, phosphatidylglycerol; MDSC, monocyte-derived myeloid-derived suppressor cells; PIP2, phosphatidylinositol (4,5)-bisphosphate; PIP3, phosphatidylinositol (3,4,5)-trisphosphate.
