# Supplementary material for: Enhanced resistance to Listeria infection in mice surviving sepsis: the role of lipid metabolism and myeloid cell reprogramming
Source: Front Pharmacol. 2025 May 30;16:1588987. doi: 10.3389/fphar.2025.1588987 (PMC12162922; doi:10.3389/fphar.2025.1588987)
Supplement: Supplementary file 2 [file DataSheet1.pdf]

# *Supplementary Material*

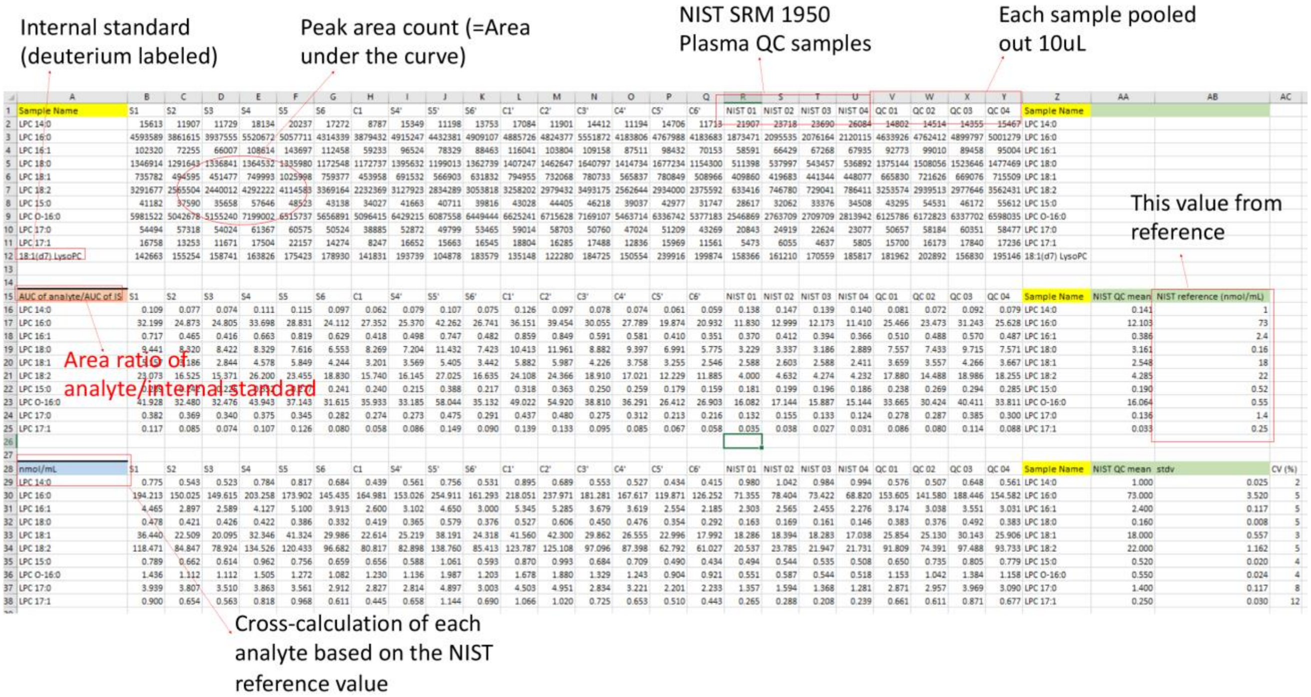


**Supplementary Figure 1. Guidance for interpreting raw lipidomics data for Supplementary Table 1.**

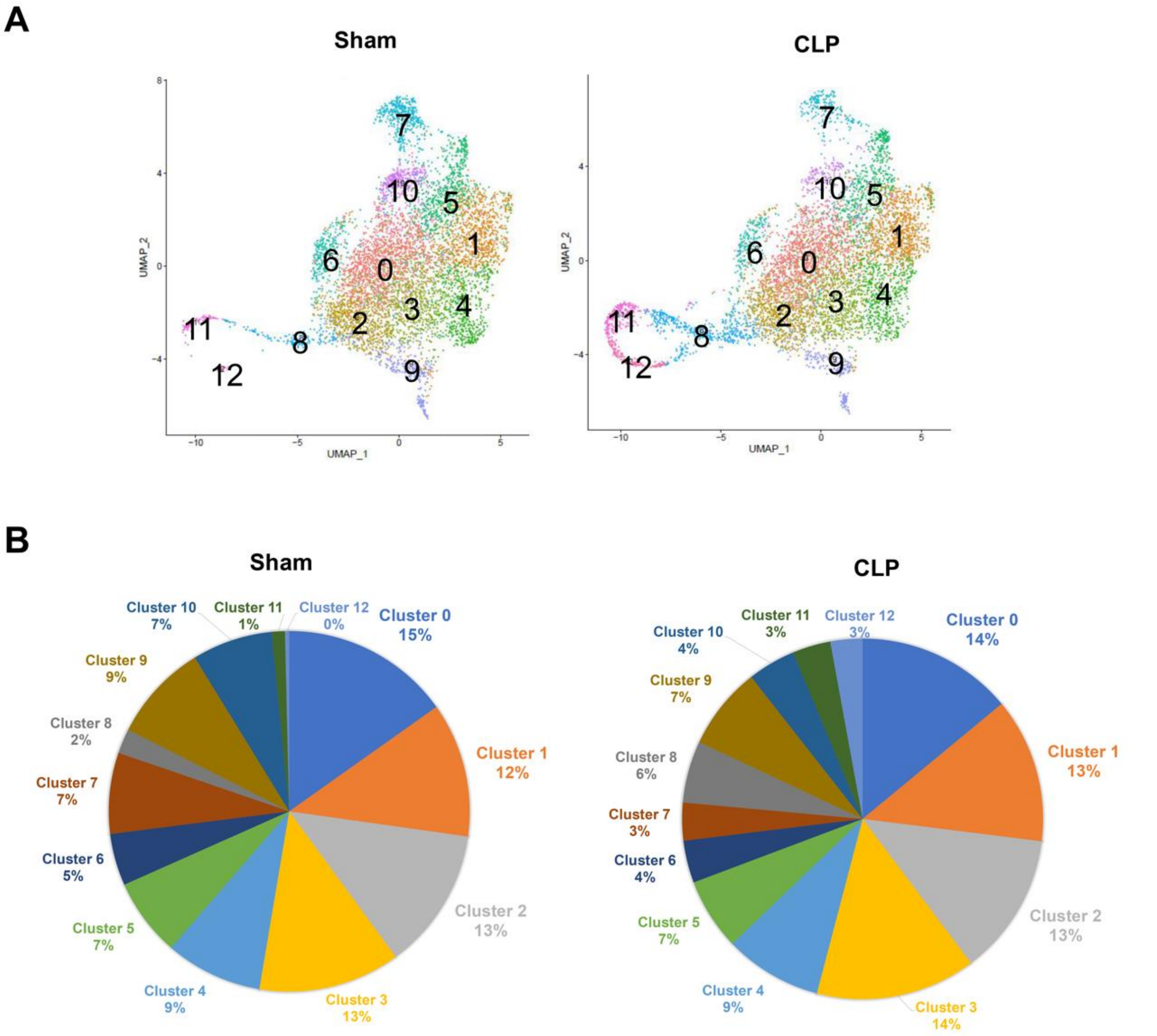


**Supplementary Figure 2. Single-Cell RNA Sequencing Classification. (A)** UMAP plot showing the clustering of single-cell CD11b+Ly6Chigh myeloid cells from sham-operated and sepsis-surviving (CLP) mice. Each point represents an individual cell, colored by cluster identity. 13 clusters were determined. Corresponding (predicting) cell type annotated based on known cell-type-specific marker genes: classical monocytes (clusters 0, 2, 3, 6), dendritic-like cells (cluster 1), M1-like macrophages (cluster 4), non-classical monocytes (clusters 5, 10), M2-like macrophage (cluster 7), monocyte-derived dendritic-like cells (cluster 9), proliferating monocytic myeloid-derived suppressor cells (M-MDSCs) (clusters 8, 11, 12). **(B)** Frequency maps indicate the distribution of 13 clusters between sham and CLP groups.

# DEGs: B6 CLP vs. sham

## CL#0

Up (P<0.01)

| logFC | AveExpr | p_val_adj | gene_names |
|---|---|---|---|
| 4.777 | 1.1953 | 0 | Lamc1 |
| 3.3617 | 1.0696 | 0.004 | Cped1 |
| 3.0356 | 0.9464 | 0.0011 | Clmn |
| 2.9642 | 1.4916 | 0.0014 | Arg2 |
| 2.9002 | 1.546 | 0.0019 | Tgm1 |
| 2.8725 | 0.3623 | 0.0053 | Pole2 |
| 2.5422 | 5.8171 | 5.43E-10 | Pbx1 |
| 2.3801 | 3.2279 | 0.0005 | Tacstd2 |
| 2.3461 | 1.1925 | 0.0042 | Mctp2 |
| 2.3374 | 1.6699 | 0.0043 | Oas1g |
| 2.2894 | 0.886 | 0.0074 | Ly6a |
| 2.2331 | 3.5525 | 0.0004 | Aig1 |
| 2.2158 | 1.5461 | 0.0057 | Nnmt |
| 2.1495 | 2.8753 | 0.0003 | Nrg1 |
| 2.1392 | 3.6358 | 0.0002 | Tarm1 |

Down (P<0.01)

| | | | |
|---|---|---|---|
| -1.9952 | 4.1836 | 4.41E-07 | Mtss1 |
| -2.1043 | 4.6271 | 0 | Ehd1 |
| -2.1557 | 0.8668 | 0.0075 | Cers4 |
| -2.1832 | 1.2257 | 0.0071 | Inpp4b |
| -2.1954 | 0.4518 | 0.0082 | Sult1a1 |
| -2.2202 | 3.3618 | 8.98E-06 | Adam19 |
| -2.2972 | 3.5221 | 6.54E-06 | Cd83 |
| -2.3121 | 1.3889 | 0.0023 | Itga8 |
| -2.356 | 1.0182 | 0.0042 | Cbr2 |
| -2.6771 | 0.7386 | 0.0018 | Pdgfb |
| -3.5745 | 5.5382 | 0.0003 | Lpl |

## CL#1

Up (P<0.01)

| logFC | AveExpr | p_val_adj | gene_names |
|---|---|---|---|
| 3.8726 | 3.2098 | 4.50E-07 | Spon1 |
| 3.6149 | 0.8552 | 0.0002 | Nrg1 |
| 3.6035 | 0.4719 | 0.0004 | Lbp |
| 3.1705 | 4.8863 | 1.46E-06 | F11r |
| 3.1296 | 2.0067 | 0.0002 | Tarm1 |
| 2.9741 | 3.3027 | 0 | Ly6a |
| 2.9143 | 0.8895 | 0.0044 | Morn2 |
| 2.617 | 2.2621 | 0.0006 | C4b |
| 2.5744 | 1.0072 | 0.0082 | Mapk13 |
| 2.5191 | 5.5631 | 5.81E-13 | Pbx1 |
| 2.1232 | 3.1511 | 0.0014 | Il21r |
| 2.0735 | 4.1188 | 9.09E-07 | Cd300lf |
| 2.0119 | 1.943 | 0.0097 | Fam234b |
| 1.8941 | 5.623 | 2.22E-11 | Vcan |
| 1.8559 | 3.1998 | 0.0033 | Ccr5 |
| 1.8556 | 4.0041 | 7E-06 | Aig1 |
| 1.6223 | 3.0803 | 0.0015 | Zdhhc23 |
| 1.6026 | 7.495 | 3.66E-14 | Ms4a6d |
| 1.571 | 4.766 | 4.50E-07 | Jaml |

Down (P<0.01)

| | | | |
|---|---|---|---|
| -1.8234 | 3.753 | 6.95E-06 | Tnip3 |
| -1.963 | 3.5662 | 2.45E-06 | Mrc1 |
| -1.966 | 2.2208 | 0.0016 | Chn2 |
| -2.0625 | 4.6 | 0 | Ehd1 |
| -2.1214 | 2.883 | 0.0001 | Heg1 |
| -2.6888 | 2.0637 | 0.0002 | Cd36 |
| -2.814 | 4.455 | 0.0001 | Cd209a |
| -2.9262 | 0.8033 | 0.0014 | Alox5 |
| -2.9482 | 0.5298 | 0.0011 | Gstp3 |
| -3.0996 | 0.2052 | 0.0014 | Efnb2 |
| -3.4948 | 0.2128 | 0.0002 | Gdf15 |
| -3.8682 | 0.2142 | 0 | Ndnf |

## CL#2

Up (P<0.01)

| logFC | AveExpr | p_val_adj | gene_names |
|---|---|---|---|
| 4.0239 | 0.8911 | 0.0004 | Cd38 |
| 3.136 | 3.7687 | 5.3E-06 | F11r |
| 2.9911 | 1.6696 | 0.0008 | Lamc1 |
| 2.4742 | 3.4678 | 0.0001 | Tarm1 |
| 2.4694 | 2.1374 | 0.0049 | Elane |
| 2.469 | 1.8779 | 0.0055 | Gpld1 |
| 2.4601 | 5.5327 | 4.26E-10 | Pbx1 |
| 2.3253 | 5.3156 | 4.86E-06 | Ier3 |
| 2.2754 | 3.7728 | 0 | Aig1 |
| 2.2352 | 3.5881 | 0.0019 | Ifitm1 |
| 2.0736 | 2.9833 | 0.0007 | Ptgfrn |
| 2.0579 | 7.99 | 4.86E-06 | Vcan |
| 2.0227 | 2.6928 | 0.0058 | Hebp1 |
| 1.973 | 5.6687 | 6.36E-08 | Gcnt2 |
| 1.9444 | 4.1567 | 0.0049 | AI506816 |
| 1.898 | 6.8224 | 7.10E-11 | Camk2d |
| 1.8852 | 4.928 | 0 | Atp11a |
| 1.8851 | 5.8334 | 6.59E-09 | Fcgr1 |

Down (P<0.01)

| | | | |
|---|---|---|---|
| -1.936 | 3.979 | 4.42E-06 | Mtss1 |
| -1.965 | 4.1792 | 0.0001 | Ehd1 |
| -1.9853 | 1.839 | 0.0058 | Hcar2 |
| -2.0009 | 3.5654 | 0 | Adam19 |
| -2.0833 | 1.5694 | 0.0067 | Il12rb2 |
| -2.1613 | 1.6026 | 0.0066 | Cgnl1 |
| -2.3008 | 1.7468 | 0.0028 | Zbtb16 |
| -2.4374 | 2.0967 | 0.0019 | Upb1 |
| -2.5382 | 1.4765 | 0.003 | Chn2 |
| -2.626 | 0.7817 | 0.0073 | Layn |
| -2.8217 | 5.5416 | 0 | Lpl |
| -2.9898 | 2.6693 | 0 | Cd83 |
| -3.2036 | 2.7795 | 0.0002 | Cd209a |

**Supplementary Figure 3. Highly upregulated and downregulated DEGs (P<0.01) of Clusters 0, 1, and 2 (CLP surviving group vs. Sham control group).**

**Supplementary Table 1. Raw lipidomics data from 6 sham (S1-S6) and 6 CLP-surviving mice (C1-C6) after 2 hours of fasting.** S1'-S6' and C1'-C1' samples are non-fasted plasma from the same animals and NIST SRM 1950 plasma QC samples. The table includes an internal standard and a reference for calculating concentration. TG, triacylglycerols, DG, diacylglycerols, and MAG, monoacylglycerols. PC, phosphatidylcholine; LPC, lyso-PC; PI, phosphatidylinositol; PS, phosphatidylserine; LP, PE, phosphatidylethanolamine; SM, sphingomyelin; FFA, free fatty acid; Cer, ceramide; HCer, hexosylceramide.

**Supplementary Table 2. Raw concentration data of lipidomics used for Figure 4.** An "A" or "B" following the name of each lipid denotes its isomeric form.
